# Anti-citrullinated protein antibody response after primary EBV infection in kidney transplant patients

**DOI:** 10.1371/journal.pone.0197219

**Published:** 2018-05-10

**Authors:** Lianne J. N. Kraal, Marieke L. Nijland, Kristine L. Germar, Dominique L. P. Baeten, Ineke J. M. ten Berge, Cynthia M. Fehres

**Affiliations:** 1 Department of Clinical Immunology and Rheumatology, Amsterdam Rheumatology and immunology Center, Academic Medical Center/ University of Amsterdam, Amsterdam, The Netherlands; 2 Renal Transplant Unit, Department of Nephrology, Department of Medicine, Academic Medical Center, University of Amsterdam, Amsterdam, The Netherlands; Rational Vaccines Inc, UNITED STATES

## Abstract

Rheumatoid arthritis (RA) is a chronic inflammatory disease of synovial joints, characterized by the presence of the highly disease-specific anti-citrullinated protein antibodies (ACPA) in approximately 70% of patients. Epstein-Barr virus (EBV) has previously been suggested to be involved in the pathophysiology of RA. However, given the high incidence of EBV in the general population and the difficulty of detecting initial infection, providing a direct link between EBV infection and RA development has remained elusive. We hypothesized that primary EBV infection may be a trigger for the development of the ACPA response *in vivo*. Using a unique cohort of 26 kidney transplant patients with a primary EBV infection, the presence of ACPA before and following infection was determined. No increase in IgG anti-CCP2 titers was detected following EBV infection. IgG anti-CCP2 antibodies were present in two patients and borderline positive in another. These three patients were HLA-DR4 negative. To test whether EBV infection may trigger a non-class switched anti-CCP2 response, IgM anti-CCP2 antibodies were analyzed. No general trend in the IgM anti-CCP2 response was observed following EBV infection. Since two out of the three IgG anti-CCP2 (borderline) positive patients were diagnosed with IgA nephropathy, 23 additional IgA nephropathy patients were tested for IgG anti-CCP2, regardless of their EBV status. All of these patients were IgG anti-CCP2 negative, indicating that IgG anti-CCP2 is not commonly present in IgA nephropathy patients. Collectively, these data do not support the hypothesis that EBV does trigger the highly RA specific ACPA response.

## Introduction

Rheumatoid arthritis (RA) is an autoimmune disease characterized by progressive symmetric poly-articular synovial joint inflammation and destruction. Anti-citrullinated protein antibodies (ACPA) can be present years before disease onset and are a very sensitive and specific biomarker[1]. In clinically overt RA, ACPA are present in 70% or greater of patients with RA and predict development of more severe bone erosions[2, 3]. The presence of these antibodies can be reliably measured using the anti-CCP2 antibody test[4]. Citrullination is a posttranslational modification involving the deimination of a positively charged arginine residue. This process is catalyzed by peptidylarginine deiminase (PAD) enzymes in the presence of calcium[5]. Genetic variants in PAD genes have been associated with a higher risk for RA[6].

Environmental factors have been linked to RA pathophysiology as well. The possible association between RA and the Epstein-Barr virus (EBV) has been extensively studied[7–9]. EBV is a human herpesvirus 4 (HHV-4), known to primarily infect B cells[10]. Most primary EBV infections occur during childhood and are asymptomatic[11]. Up to 90% of the adult world population is infected[12]. Compared to non-RA EBV seropositive controls, RA patients have higher titers of EBV antibodies in serum, a higher load of EBV DNA, more EBV-infected B cells in the peripheral blood and an increased frequency of circulating EBV-specific CD8^+^ T cells, suggesting that there might be a link between RA and EBV[9, 13]. However, several other studies did not show a sero-epidemiological association between RA and EBV (reviewed in[7]).

Several lines of evidence indicate that EBV infection may influence the balance between tolerance and autoimmunity. Viral proteins, like latent membrane protein 1 (LMP1) and latent membrane protein 2A (LMP2A), are produced by the EBV-infected B cell to modulate the immune response[14–18]. In order to survive, proliferate and become activated, B cells need stimulation via several receptors, like the B-cell receptor (BCR) and CD40[19]. Stimulation of these receptors also induces negative regulatory pathways, which limit and terminate the immune response[20]. Depending on the strength of the signal through the BCR, autoreactive cells are eliminated, subjected to receptor editing, or rendered functionally anergic[21, 22]. However, apoptosis of germinal center B cells with an autoreactive B-cell receptor (BCR) can be prevented by EBV[23, 24]. The EBV viral protein LMP2A simulates an activated BCR and therefore provides a continuing survival signal. The EBV viral protein LMP1 resembles CD40 in function and effect on B cells[25]. Although CD40 needs binding to its ligand CD154, which is expressed primarily on activated T cells, LMP1 can activate the downstream pathways in a CD154—and therefore T-cell—independent manner[18]. Moreover, LMP1 cannot, like CD40, downregulate TRAF expression upon stimulation, implicating an on-going immune response[26, 27]. LMP1 also upregulates the expression of the apoptosis-inhibiting proteins B cell lymphoma (Bcl)-2 and A20[12, 28]. Moreover, LMP1 can upregulate activation-induced cytidine deiminase (AID) expression, thereby stimulating somatic hypermutation (SHM)[18]. Via LMP1 and LMP2A, EBV can thus stimulate the selection of low-affinity antibody-producing B cells, including autoreactive B cells, which otherwise should be excluded of the immune system[29].

EBV has also been linked specifically to the APCA response. One study showed that an EBV-transformed lymphoblastoid cell line contained citrullinated EBNA1[8]. Other studies show recognition of citrullinated epitopes of EBNA1 by RA serum, with ACPA being cross-reactive with anti-citrullinated EBNA1 antibodies[8, 30].[8, 30]. Also, EBV infection has been shown in 70% of the ACPA-producing plasma cells in the vicinity of ectopic lymphoid neogenesis (ELN) tissue in the rheumatoid synovium[31].

Based on this knowledge we hypothesized that primary EBV infection may be a trigger for the development of the ACPA response *in vivo*. Investigating a possible link *in vivo* is complicated by the fact that around 90% of the world population is EBV infected, and the precise moment of primary EBV-infection is usually not recognized since it occurs most often in childhood and is mostly asymptomatic[12]. We used a unique cohort of EBV-negative kidney transplant patients of whom blood was collected before and after transplantation with an EBV-positive kidney, to be able to investigate if primary EBV infection, caused by the kidney transplantation, is a trigger for the development of the ACPA response *in vivo*. Since EBV status is tested before and after transplantation in kidney transplant recipients, this patient group provided a unique opportunity to investigate the hypothesized transient ACPA response after primary EBV infection.

## Materials and methods

### Patients and samples

We included 26 patients that developed a primary EBV infection after transplantation of an EBV+ kidney. These 26 patients were selected from a large longitudinal cohort of kidney transplant patients, collected by the Renal Transplant Unit of the Department of Nephrology in the AMC, based on development of a primary EBV infection post-transplantation and presence of serum samples pre- and post-infection. Kidney transplantation patients were regularly tested for EBV infection after transplantation, both by serology and qPCR. When either serology (VCA IgG and IgM and EBV anti-EBNA IgG) or qPCR (EBNA1 gene) turned out positive, the patient was considered to be EBV positive and was included in our study.

To investigate the possible induction of an ACPA response after primary EBV infection, ACPA levels were tested at three time points. The first samples from EBV negative individuals were available at a median of 4 months (range 0–36 months) before or at the day of the kidney transplantation and subsequent start of immunosuppressive drugs. All donor kidneys came from EBV seropositive donors. Following transplantation, the patients were monitored for EBV infection. The second serum sample included in this study is from the first moment of EBV detection, median 13 weeks (range 6–154 weeks) after kidney transplantation. At this point all patients used immunosuppressive drugs, with the majority of them using mycophenolate mofetil, tacrolimus and prednisolone. As we hypothesized the ACPA response to be transient, our third time point was the next moment of sample drawing. This was 1 month (median, range 0–34 months) after EBV infection. At this time point, patients were still on the same immunosuppressive drug regimen.

Patients gave their written informed consent. The Medical Ethical Committee of the Academic Medical Center Amsterdam, The Netherlands, approved all procedures.

### Virological analyses

To determine the EBV viral load, quantitative polymerase chain reaction (qPCR) for EBV was performed. Total RNA was extracted from blood samples and reverse transcribed according to the manufacturer’s instructions. qPCR was performed using TagMan gene expression assays. The following primers were used (5’- 3’): EBV forward CACAATGTCGTCTTACACCATTGA and EBV reverse AGGTCCTTAATCGCATCCTTCA. As detection probe CGTCTCCCCTTTGGAATGGCCC was used.

EBV serostatus was determined by qualitative measurement of specific IgG against the viral capsid antigen (VCA) and against EBV nuclear antigen using the anti-EBV VCA IgG enzyme-linked immunosorbent assay (ELISA), the anti-EBV nuclear antigen ELISA (Bio-Rad Medical Diagnostics, Dreieich, Germany) or using the LIAISON® EBV IgM, VCA IgG and EBNA IgG chemiluminescence immunoassays (DiaSorin S.p.A., Italy). All tests were performed according to the instructions of the manufacturers.

### Anti-CCP2 antibody ELISA

Serum was tested for the presence of IgG anti-CCP2 antibodies using a commercial ELISA kit (IMMUNOSCAN CCPlus, Euro Diagnostica, Sweden)[32]. Antibody level is expressed in arbitrary units (U/ml), after reference to standard. Antibody levels above 25 AU/mL are considered positive. IgM anti-CCP2 antibodies were detected using the previously mentioned IgG anti-CCP2 antibody ELISA. Instead of the IgG streptavidin from the IMMUNOSCAN CCPlus kit, horseradish peroxidase (HRP)-conjugated goat anti-human IgM (Jackson Immuno-Research Laboratories inc.) was used. A serial dilution of IgM anti-CCP2 positive serum was used as standard. Results are expressed in U/ml, these units were arbitrary. The cutoff value was 210 U/mL for the negative control (determined using serum of healthy controls) and the positive control was 527 U/mL. To test for specificity, an ELISA using the control arginine cyclic peptide was performed as described previously[33].

## Results

### Antibody response to EBV and EBV-viral load

In this study, we aimed to address the question whether a primary EBV infection could induce a transient ACPA response. To this end, we included 26 patients that developed a primary EBV infection after kidney transplantation and analyzed the development of a CCP2 response over time. The patient characteristics are shown in Table 1.

**Table 1:** Patient characteristics kidney transplant patients with a primary EBV infection.

	n = 26
**Gender, male, n (%)**	18 (69)
**Age, median (range), years**	37 (19–77)
**HLA-DR4 positivity, n (%)**	7 (27)
**Smoking history, n (%)**	9 (38)
**Time point 1 (months before the first time point with positive EBV qPCR or serology, i.e. time point 2), median (range)**	3 (1–35)
**Time point 3 (months after the first time point with positive EBV qPCR or serology, i.e. time point 2), median (range)**	1 (0–34)

In order to detect the primary EBV infection after transplantation of the kidney, we determined the presence of anti-EBV antibody responses and measured the EBV viral load. Viral capsid antigen (VCA) IgG and IgM and EBV anti-EBNA IgG were measured in all 26 patients; the EBV-viral load was determined by qPCR in 25 out of the 26 patients. In eight of the 26 patients serology was tested positive first, followed by a positive viral load in six of the eight patients. In the two remaining patients a positive serology was not followed by a positive viral load, possibly due to that fact that the viral load is high during the lytic infection which only lasts for approximately ten days[34]. In sixteen patients a positive viral load was detected first, followed by positive serology in thirteen of them; three patients remained seronegative, probably due to the fact that the moments of blood drawing were not frequent enough to remain in the correct time frame. In two patients viral load and serologic markers were tested positive at the same time. Table 2 shows the distribution of the responses to the specific antibodies. The anti-EBV response per individual patient is depicted in S1 Table.

**Table 2:** EBV-antibody responses and EBV viral load detection by qPCR.

	Positive	Negative
**VCA-IgM, n**	12/26	14/26
**VCA-IgG, n**	21/26	3/26*
**Anti-EBNA IgG, n**	8/26	18/26
**qPCR (EBNA1 gene), n**	23/25	2/25**

*These three patients never had any antibody response against EBV, only qPCR was positive.

** In these two patients a positive EBV viral load was never detected, although they did show conversion to positive EBV serology.

### IgG anti-CCP2 response after primary EBV infection

To test whether EBV infection does induce a maturated, class-switched ACPA response *in vivo*, we tested serum samples from 26 kidney transplant patients that developed a primary EBV infection for the presence of IgG anti-CCP2 antibodies before and shortly after EBV infection.

**Fig 1:**
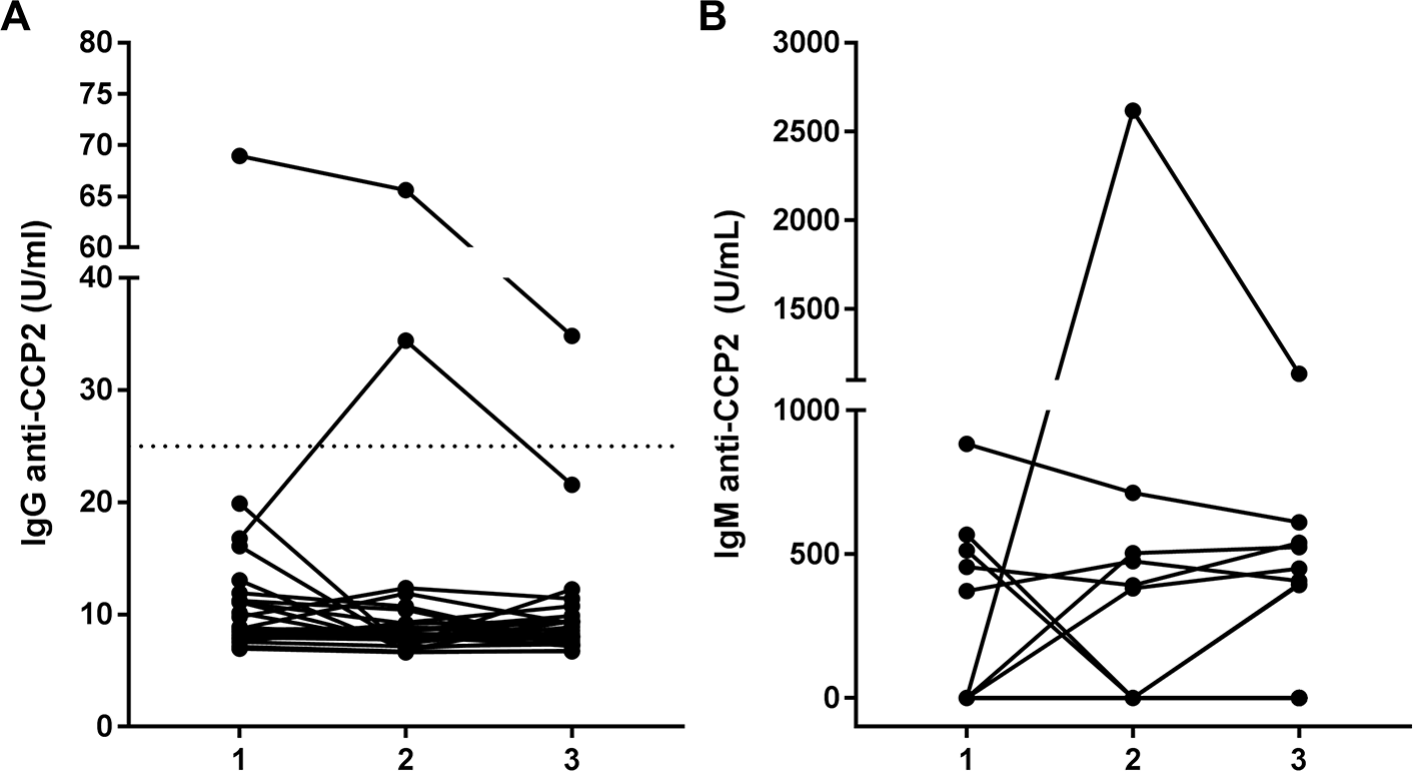
IgG and IgM anti-CCP2 antibody expression in serum. Time points are (1) the first available time point before EBV infection, (2) the first time point with a positive EBV viral load or serology, (3) the first available time point after time point 2. (A) IgG anti-CCP2 antibody levels of all tested patients (n = 26) in arbitrary units as defined by the IMMUNOSCAN CCPlus test. Values above 25 U/ml, represented by a dotted line, are considered to be positive. (B) IgM anti-CCP2 antibody levels of all tested patients (n = 26) in arbitrary units.

At baseline, before EBV infection, the median IgG anti-CCP2 titer was 9 U/mL (range 7–69 U/mL). The median titer was not changed directly after EBV infection (titer 8 U/mL, range 7–66 U/mL) and at a median of 24 weeks (range 1–35 weeks) after EBV infection (median of 8 U/mL, range 7–35 U/mL). In 23 out of 26 patients, no anti-CCP2 IgG above 25 U/mL was detected at any time. Two patients had a positive anti-CCP2 IgG titer (defined as >25 U/ml). The first patient had a positive IgG anti-CCP2 titer before transplantation (69 U/mL). The patient was diagnosed with IgA nephropathy, HLA-DR8-12 positive and did not smoke. Immediately after infection, six weeks after kidney transplantation, the IgG anti-CCP2 titer remained stable (65 U/mL), but declined to 35 U/mL in one week (Fig 1A). The second patient was IgG anti-CCP2 negative before transplantation and EBV infection, but became IgG anti-CCP2 positive (34 U/mL) after EBV infection, which occurred twelve months after kidney transplantation. Eight months later, the IgG anti-CCP2 titer had declined to 22 U/mL (Fig 1A). SLE had caused this patient’s renal insufficiency, which was clinically silent from one year before transplantation. Her HLA-DR typing was HLA-DR11(5)-10 and she did not smoke. Another patient had a borderline IgG anti-CCP2 titer of 20 U/mL before transplantation (data not shown). This patient was diagnosed with IgA nephropathy, HLA-DR13(6)-7 positive and did smoke. At the moment of EBV infection, almost twelve months after kidney transplantation, and the subsequent time point, almost 6 months later, no IgG anti-CCP2 was detected.

To exclude the possibility that we could not detect an IgG anti-CCP2 response in the kidney transplants patients because those patients receive immunosuppressive drugs that might interfere with the development of a mature antibody response, we compared anti-EBV antibody levels measured at the third time point (median 1 month after EBV infection, range 0–34 months) in serum of the kidney transplant patients with anti-EBV antibody levels detected in the non-immunosuppressed kidney donors (S1 Fig). No significant differences were observed in the VCA-IgG and EBNA-IgG levels between the kidney transplant patients and the donors, indicating that the kidney transplant patients were able to develop a mature antibody response despite the treatment with immunosuppressive drugs (S1 Fig).

Collectively, no trend towards an increase in IgG anti-CCP2 titers was observed following EBV infection. Therefore, these data do not support our hypothesis that EBV primo-infection can induce a transient ACPA response.

### IgM anti-CCP2 response after primary EBV infection

As EBV may trigger an ACPA response but other factors may be needed for its maturation to a full-blown, affinity-matured IgG ACPA response, we tested for the presence of IgM anti-CCP2 as well.

The median IgM anti-CCP2 titer at baseline, before EBV infection, was 237 U/mL (range 0–885 U/mL). The titer remained stable shortly after EBV infection (341 U/mL, range 0–2617 U/mL) and at the subsequent time point, at a median of six weeks later (range 1–91 weeks) with a median titer of 272 U/mL (range 0–1136 U/mL). Ten out of 26 patients had a detectable IgM anti-CCP2 titer at any time point (Fig 1B, S3 Table). In five patients, IgM anti-CCP2 antibodies were present before EBV infection, with titers ranging from 372 to 885 U/mL. In three of these patients the titer remained stable. These patients were diagnosed with membranoproliferative glomerulonephritis type 2, hemolytic uremic syndrome, and SLE (the same patient with positive IgG anti-CCP2), respectively. None of these patients was HLA-DR4 positive. IgM anti-CCP2 antibodies declined to below detectable levels after kidney transplantation and the start of immunosuppressive drugs in the two other patients. These patients were diagnosed with Lent syndrome and IgA nephropathy (the same patient with positive IgG anti-CCP2), respectively. They did not have a smoking history and were HLA-DR4 negative. In three patients, IgM anti-CCP2 antibodies appeared directly after primary EBV infection, with titers of 381 U/mL, 2617 U/mL and 504 U/mL respectively. The diagnoses were focal segmental glomerulosclerosis, kidney damage due to vesico-urethral reflux and hypertension, and unknown, respectively. The titers remained stable and relatively high in all three patients over time. No smoking history or HLA-DR4 positivity was recorded for these patients. Finally, in two other patients, positive IgM anti-CCP2 titers (393 U/mL and 396 U/mL) were only detected at time point three (70 respectively six weeks after EBV infection); one patient was diagnosed with agenesis of the left side kidney and a hypoplastic kidney on the right side and was HLA-DR3-4 positive, the other was diagnosed with essential hypertension with secondary focal segmental glomerulosclerosis and was HLA-DR2-6 positive.

In summary, ten out of 26 patients were IgM anti-CCP2 positive at some time point, with three patients showing a clear increase in IgM anti-CCP2 titers after EBV primo-infection.

### Specificity of the IgG and IgM anti-CCP2 antibodies

**Fig 2:**
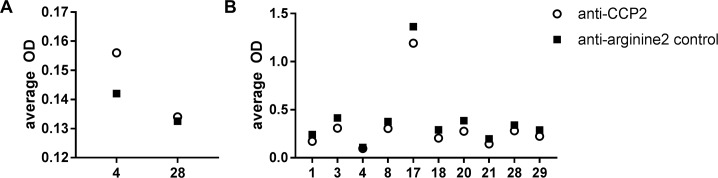
OD values for the IgG and IgM anti-CCP2 antibodies compared to the IgG and IgM anti-arginine2 antibody controls. Patients previously tested positive for IgG (n = 2) or IgM anti-CCP2 (n = 10) antibodies were additionally tested for IgG respectively IgM anti-arginine2 antibodies. (A) IgG anti-CCP2 and IgG anti-arginine2 antibody levels. (B) IgM anti-CCP2 and IgM anti-arginine2 antibody levels. Patient numbers are depicted on the x-axis.

To test whether the citrulline reactivity in the IgG anti-CCP2 positive samples (two patients) and IgM anti-CCP2 positive samples (ten patients) were specific for citrulline, we tested the samples for reactivity against the arginine 2 control peptide (CArgP2). All tested samples did show a signal for reactivity against the CArgP2 peptide, with similar intensity as the signal for reactivity to the CCP2 peptide, indicating that the anti-CCP2 response is not specific (Fig 2).

### Anti-CCP2 antibodies in IgA nephropathy patients

As one of the patients with a positive IgG anti-CCP2 and the patient with borderline IgG anti-CCP2 levels at baseline were both diagnosed with IgA nephropathy and as IgA nephropathy has been reported to occur more frequently in patients with inflammatory diseases such as spondyloarthritis, we were interested whether the presence of low titer ACPA is a common feature of patients with IgA nephropathy[35, 36]. Baseline samples from 23 additional IgA nephropathy patients were tested for IgG anti-CCP2. These patients were all EBV positive before kidney transplantation. The patient characteristics are shown in Table 3, more detailed information per patient is provided in S2 Table.

**Table 3:** Characteristics of the additional IgA nephropathy patients.

	n = 23
**Gender, male, n (%)**	22 (96)
**Age, years, median (range)**	45 (27–68)
**HLA-DR4 positivity, n (%)**	11 (48)
**Smoking history, n (%)**	5 (28)
**Time before or after kidney transplantation,** **weeks, median (range)**	0 (-267–24)

Blood samples were drawn from 267 weeks before to 24 weeks after kidney transplantation (median 0 weeks). Since in the majority of patients the moment of blood drawing was before kidney transplantation, most of the patients (21 out of 23 patients) did not yet use immunosuppressive drugs. None of these additional patients had detectable ACPA IgG serum levels (data not shown).

## Discussion

Although some studies point towards a role for EBV in the RA pathophysiology, nothing is yet known about the effect of primo EBV infection on the ACPA response *in vivo*. Since EBV does have prevalence up to 90% in the general population, with an asymptomatic infection mainly occurring during childhood, studying the effects of a primary EBV infection on ACPA levels is challenging[11, 12]. In this study, we tested the hypothesis that EBV primo-infection triggers ACPA responses by measuring IgG and IgM anti-CCP2 in serum of kidney transplant patients with a primary EBV infection. We observed no increase in citrulline-specific IgG or IgM anti-CCP2 antibody levels after primary EBV infection in kidney transplant patients. One clearly positive IgG anti-CCP2 titer was observed at baseline in an IgA nephropathy patient. This positivity disappeared after transplantation and subsequent start of immunosuppressive drugs. Another patient, diagnosed with SLE, became ACPA positive following EBV infection, with the positivity disappearing within eight months. Also, another IgA nephropathy patient in this cohort showed a borderline positive IgG anti-CCP2 titer (20 U/mL) before transplantation. However, a correlation between IgA nephropathy and IgG anti-CCP2 positivity was not confirmed in an independent cohort of IgA nephropathy patients. Moreover, all IgG and IgM CCP2 positive samples did also show a positive signal against the CArgP2 peptide, indicating that the anti-CCP2 response was not citrulline specific. False-positive ACPA reactivity has been described in other non-rheumatic conditions, such as autoimmune hepatitis, tuberculosis and in patients with *Leishmania donovani* infection[37]. In the hepatitis study CCP2 specific responses were observed in half of the hepatitis samples[38], whereas only 22% of the sera from pulmonary tuberculosis patients was citrulline specific for CCP1 peptide compared to 94% of the sera from RA patients[39]. As we cannot detect a citrulline-specific response in the CCP2 ELISA, which recognizes a broad repertoire of citrullinated epitopes, we argue that, albeit not formally excluded, it is unlikely that there would be other, very specific anti-citrulline reactivities.

As EBV may trigger an ACPA response, but other factors may be needed for its maturation to a full-blown, affinity-matured IgG ACPA response, we have tested for the presence of IgM anti-CCP2. It has been shown that ACPA IgM responses are detectable in RA patients and are stable over time[40]. Recognition of defined citrullinated antigens by IgM ACPA was restricted to samples that also displayed recognition by IgG ACPA, but the IgM ACPA response showed a more restricted antigen recognition profile than IgG ACPA.[40] Moreover, no IgM CCP2 responses were observed in IgG CCP2 negative RA patients[41] or in healthy controls[42], suggesting that IgM CCP2 responses are rather specific for ACPA positive RA patients. Although we observed detectable IgM anti-CCP2 titers in 10 out of 26 patients, we concluded that EBV does not induce a transient ACPA IgM response based on three observations: 1. all IgM CCP2 positive samples did also test positive against the CArgP2 peptide; 2. we did not observe a pattern between IgM CCP2 responses and EBV infection in time, as 5 patients already showed IgM CCP2 reactivity observed prior to EBV infection; and 3. we could not detect a connection between the IgM CCP2 positive patients and the IgG CCP2 patients. Collectively, our data do not provide evidence for our hypothesis that EBV induces a transient ACPA response.

When interpreting these data, two major limitations should be kept in mind. Firstly, following kidney transplantation, all patients were prescribed immunosuppressive drugs, aiming to effectively blunt the onset of alloimmune responses. These drugs may equally affect autoimmune responses and the influence of the immunosuppressants on the ACPA titers may be different in the initiation and/or amplification phase of the response. It has been described that the humoral antibody response is suppressed in patients using immunosuppressive drugs, with the most distinct suppression by mycophenolate mofetil and also some suppression by cyclosporine A[43–45]. Since these immunosuppressive drugs were used by most of the patients in our cohort, this could be an explanation for the decline in ACPA levels after kidney transplantation and subsequent EBV infection, which we observed in a patient diagnosed with IgA nephropathy. However, it should be stated that the majority of these patients did still produce anti-EBV antibodies in a range similar to that of the kidney donors, which were not treated with immunosuppressive drugs.

The second limitation concerns the limited size of the current sample set. Given the rarity of primary EBV infection during adulthood and the difficulties of gathering samples of primary EBV infection infected patients, we could not include more than 26 patients. We did not observe the hypothesized effect of a primary EBV infection, namely a transient ACPA response, in this cohort. The ACPA response however may require additional genetic and environmental factors. The contribution of genetic factors to the development of RA is estimated around 60%, of which one third from genes on the MHC class II locus[46, 47]. In particular HLA-DR4 is associated with ACPA positive RA[48, 49]. In our cohort, however, only seven patients did have an HLA-DR4 haplotype. It could be that we were unable to detect an IgG anti-CCP2 antibody response, since development towards an IgG response requires T-cell help. These antigen-specific T cells require a specific haplotype context. Moreover, evidence points towards a strong gene-environment interaction with smoking in the onset of especially ACPA positive RA[50]. In our cohort, only 38% of the included patients had a history of smoking. It should also be noted that determining EBV infection and viral load in kidney transplant patients is complicated by an atypical EBV gene expression pattern in immunosuppressed patients[51]. The one SLE patient in our cohort had positive IgG and IgM anti-CCP2 titers. It has previously been described that ACPA can be present in SLE patients[52].

Our data do not exclude another role for EBV in the humoral autoimmune responses in RA. EBV could, for example, play a role not during primo infection but during its chronic phase. This could be related to autonomous survival of autoimmune B cells. For example, EBV can stimulate the outgrowth of autoreactive B cells[23, 24], enhance B cell survival by providing a continue survival signal[15, 18] and modulate the immune response, using its viral proteins LMP1 and LMP2A[14–18].

## Supporting information

S1 Table Characteristics of the kidney transplant patients with a primary EBV infection. (DOCX)

S2 Table Characteristics of the additional IgA nephropathy patients. (DOCX)

S3 Table IgM anti-CCP2 levels per time point. (DOCX)

S1 Fig Anti-EBV antibody levels measured in kidney transplant patients and kidney donors. (DOCX)
