# Supplementary material for: Anti-citrullinated protein antibody response after primary EBV infection in kidney transplant patients
Source: PLoS One. 2018 May 10;13(5):e0197219. doi: 10.1371/journal.pone.0197219 (PMC5945038; doi:10.1371/journal.pone.0197219)
Supplement: S1 Table — (DOCX) [file pone.0197219.s001.docx]

**S1 Table. Characteristics of the kidney transplant patients with a primo-EBV infection**

| **Patient number** | **IgM status** | **Time points with IgM positivity** | **IgG status** | **Time points with IgG positivity** | **Underlying disease** | **Age at time of transplantation** | **Gender** | **Medication** | **HLA-DR** | **Smoking history** | **Time point 1 (months)** | **Time point 3 (months)** | **VCA IgM** | **VCA IgG** | **Anti-EBNA IgG** | **EBV viral load (peak)** |
| --- | --- | --- | --- | --- | --- | --- | --- | --- | --- | --- | --- | --- | --- | --- | --- | --- |
| 1 | + | 3 | - | none | Agenesis left kidney, hypoplastic kidney right | 26 | M | 1,2,3 | 3-4 | Unknown | 12 | 11 | + | + | - | 8200 |
| 2 | - | none | - | none | Focal glomerulosclerosis | 22 | M | 3,5 |  | Yes | 1 | 34 | + | + | + | 26500 |
| 3 | + | 1 | - | none | Lent syndrome (agenesis left kidney, dysplastic kidney right) | 35 | M | 1,2,3,4 | 17-7 | No | 5 | 0 | + | +/- | - | 1450 |
| 4 | + | 1 | + | 1,2,3 | IgA nephropathy | 29 | M | 1,2,3,4 | 8-12 | No | 10 | 0 | +/- | + | - | 1000 |
| 5 | - | none | - | none | Hypertensive nephropathy | 77 | M | 1,2,3,4 | 1-4, 53 | Yes | 3 | 0 | - | + | - | 1400 |
| 6 | - | none | - | none | Familiar cystic kidney disease | 48 | M | 2,3 | 3-13 | No | 35 | 11 | - | + | - |  |
| 7 | - | none | - | none | Focal glomerulosclerosis | 59 | M | 1,2,3,4 | 4-11 | Yes | 2 | 0 | - | - | - | 1000 |
| 8 | + | 2,3 | - | none | Unknown | 33 | F | 1,2,3,4 | 7-10 | No | 3 | 1 | - | + | + | 3620 |
| 9 | - | none | - | none | Morbus Alport | 25 | M | 1,3,5 | 1-3 | Yes | 5 | 0 | + | + | - | 1500 |
| 12 | - | none | - | none | Post-anoxic acute tubulus necrosis at birth | 25 | M | 1,2,3,4 |  | No | 1 | 1 | + | + | - | 1000 |
| 13 | - | none | - | none | Pauci-immune extracapillar glomerulonephritis  (positive MPO ANCA) | 62 | M | 1,2,3 | 1 | Yes | 2 | 0 | +/- | + | - | 15800 |
| 14 | - | none | - | none | Familiar cystic kidney disease | 51 | F | 1,2,4,6 | 17-13 | Yes | 6 | 13 | - | + | + | 0 |
| 15 | - | none | - | none | Membranous glomerulopathy | 70 | M | 1,2,3 | 17-14, 53 | Unknown | 1 | 0 | - | - | - | 193000 |
| 16 | - | none | - | none | Contracted kidney right side, hypertensive damage kidney left side | 27 | F | 1,2,3 | 4-13 | No | 5 | `0 | - | - | - | 2260 |
| 17 | + | 2,3 | - | none | Focal segmental glomerulosclerosis, idiopatic renal failure | 38 | M | 1,3,5 | 3-12 | No | 2 | 0 | + | + | + | 1500 |
| 18 | + | 1,2,3 | - | none | Membranoproliferative glomerulonephritis type II  (dens deposit disease) | 49 | M | 1,3,4,5 | 10-14 | No | 1 | 1 | + | + | - | 28000 |
| 19 | - | none | - | none | Reflux nephropathy | 19 | M | 1,2,4,6 | 1-4, 53 | No | 12 | 2 | - | + | - | 4390 |
| 20 | + | 1,2,3 | - | none | Hemolytic uremic syndrome | 21 | F | 1,3,5 | 15-5, 51-53 | No | 35 | 10 | + | + | +/- | 10400 |
| 21 | + | 2,3 | - | none | Vesico-urethral reflux, hypertensive damage | 30 | F | 1,2,3,4 | 13-7 | No | 2 | 1 | - | + | - | 120000 |
| 22 | - | none | - | none | Focal glomerulosclerosis | 41 | M | 3,4,5,7 | 17-4 | No | 1 | 0 | - | + | + | 1000 |
| 23 | - | none | - | none | Familiar cystic kidney disease | 57 | F | 3,4,5,7 | 17-13 | Yes | 3 | 0 | + | + | + | 16100000 |
| 25 | - | none | - | none | Contracted kidneys both sides and recurrent urinary infections | 47 | M | 1,3,4,5,6 | 1-13(6) | Yes | 11 | 2 | - | + | - | 11300 |
| 26 | - | none | +/- | 1 | IgA nephropathy, acute tubulus necrosis | 41 | M | 1,2,3 | 13(6)-7 | No | 9 | 5 | - | + | - | 2820 |
| 27 | - | none | - | none | Dysplastic kidneys | 20 | M | 1,2,3,4 | 7-51 | No | 2 | 0 | + | + | - | 2720000 |
| 28 | + | 1,2,3 | + | 2 | SLE | 20 | F | 1,2,3,4 | 11(5)-10 | No | 12 | 8 | - | + | + | 0 |
| 29 | + | 3 | - | none | Essential hypertension with secondary focal segmental glomerulosclerosis | 54 | F | 1,2,3 | 2-6 | Yes | 1 | 1 | - | + | - | 27300 |

**Legenda**

1: mycophenolate mofetil (cellcept)

2: tacrolimus (prograft, advagraf)

3: corticosteroids (prednisolon)

4: basiliximab (simulect)

5: cyclosporin (neoral, sandimmune)

6: decay accelerating factor (DAF)

7: mycophenolzuur (myfortic)

8: azathioprine

Time point 1: months before time point 2

Time point 2: the first time point with a positive EBV qPCR or serology, t=0

Time point 3: months after time point 2

IgM and IgG do refer to IgM anti-CCP2 and IgG anti-CCP2 positivity
