# Supplementary material for: Anti-citrullinated protein antibody response after primary EBV infection in kidney transplant patients
Source: PLoS One. 2018 May 10;13(5):e0197219. doi: 10.1371/journal.pone.0197219 (PMC5945038; doi:10.1371/journal.pone.0197219)
Supplement: S2 Table — (DOCX) [file pone.0197219.s002.docx]

**S2 Table. Characteristics of the additional IgA nephropathy patients**

| **Patient number** | **Age at moment of**  **sample drawing** | **Gender** | **Medication** | **HLA-DR** | **Smoking history** | **Sample drawing before or after transplantation (weeks)** |
| --- | --- | --- | --- | --- | --- | --- |
| IgA1 | 63 | M | 3,4,5 | 4,9 | No | 0 |
| IgA2 | 53 | M | 1,3,5 | 4,13(6) | No | 1 |
| IgA3* | 59 | M | none | 15(2),7 | No | 0 |
| IgA4 | 60 | M | 3,8 | 3,4 | Unknown | -267 |
| IgA5 | 62 | M | 1,2,3,4 | 4,8 | No | 0 |
| IgA6 | 35 | F | 1,3,5 | 11(5),11(5) | Yes | -43 |
| IgA7 | 52 | M | 1,2,3,4 | 1,7 | Unknown | 0 |
| IgA8 | 54 | M | 1,3,5 | 15(2),4 | No | 0 |
| IgA9 | 40 | M | 1,2,3,4 | 13(5) | No | 24 |
| IgA10 | 31 | M | 1,3,5 | 11(5),11(5) | No | 0 |
| IgA11 | 39 | M | 1,3,5 | 16(2),7 | Yes | 0 |
| IgA12 | 34 | M | 1,2,3,4 | 4,14(6) | Yes | 0 |
| IgA13 | 45 | M | 1,3,5 | 1,4 | No | 0 |
| IgA14 | 52 | M | 1,3,5 | 3,8 | Yes | 0 |
| IgA15 | 38 | M | 1,2,3,4 | 1,8 | Unknown | 0 |
| IgA16 | 27 | M | 1,2,3 | 14(6),4 | No | 0 |
| IgA17 | 52 | M | 1,2,3,4 | 1,13(6) | Unknown | 0 |
| IgA18 | 30 | M | 3,4,5,7 | 4,13(6) | Unknown | 0 |
| IgA19 | 44 | M | 3,5,7 | 11(5),13(6) | No | 0 |
| IgA20 | 68 | M | 2,3,4,5 | 4,13(6) | No | 1 |
| IgA21 | 42 | M | 2,3,4,5 | 1,13(6) | No | 0 |
| IgA22 | 53 | M | 3,4,5,7 | 4,11(5) | No | 0 |
| IgA23 | 42 | M | 1,2,3,4 | 15(2),7 | yes | -157 |

*IgA3: Donor kidney was removed days after the transplantation so no further medical treatment needed.

**Legenda**

1: mycophenolate mofetil (cellcept)

2: tacrolimus (prograft, advagraf)

3: corticosteroids (prednisolon)

4: basiliximab (simulect)

5: cyclosporin (neoral, sandimmune)

6: decay accelerating factor (DAF)

7: mycophenolzuur (myfortic)

8: azathioprine

Time point 1: months before time point 2

Time point 2: the first time point with a positive EBV qPCR or serology, t=0

Time point 3: months after time point 2

IgM and IgG do refer to IgM anti-CCP2 and IgG anti-CCP2 positivity
