# Supplementary material for: Anti-citrullinated protein antibody response after primary EBV infection in kidney transplant patients
Source: PLoS One. 2018 May 10;13(5):e0197219. doi: 10.1371/journal.pone.0197219 (PMC5945038; doi:10.1371/journal.pone.0197219)
Supplement: S3 Table — (DOCX) [file pone.0197219.s003.docx]

**S3 Table. IgM anti-CCP2 levels per time point**

Only IgM anti-CCP2 positive patients are shown.

| **Patient number** | **IgM in U/mL,**  **time point 1** | **IgM in U/mL,**  **time point 2** | **IgM in U/mL,**  **time point 3** |
| --- | --- | --- | --- |
| 1 | neg | neg | 393 |
| 3 | 513 | neg | neg |
| 4 | 568 | neg | neg |
| 8 | neg | 381 | 450 |
| 17 | neg | 2617 | 1136 |
| 18 | 372 | 476 | 406 |
| 20 | 885 | 714 | 611 |
| 21 | neg | 504 | 524 |
| 28 | 456 | 392 | 540 |
| 29 | neg | neg | 396 |
