# Supplementary material for: Anti-citrullinated protein antibody response after primary EBV infection in kidney transplant patients
Source: PLoS One. 2018 May 10;13(5):e0197219. doi: 10.1371/journal.pone.0197219 (PMC5945038; doi:10.1371/journal.pone.0197219)
Supplement: S1 Fig — (DOCX) [file pone.0197219.s004.docx]

**S1 Fig. Anti-EBV antibody levels measured in kidney transplant patients and kidney donors.**
